# Impact of SSTR PET on Inter-Observer Variability of Target Delineation of Meningioma and the Possibility of Using Threshold-Based Segmentations in Radiation Oncology

**DOI:** 10.3390/cancers14184435

**Published:** 2022-09-13

**Authors:** Florian Kriwanek, Leo Ulbrich, Wolfgang Lechner, Carola Lütgendorf-Caucig, Stefan Konrad, Cora Waldstein, Harald Herrmann, Dietmar Georg, Joachim Widder, Tatjana Traub-Weidinger, Ivo Rausch

**Affiliations:** 1 Division of Nuclear Medicine, Department of Biomedical Imaging and Image Guided Therapy, Medical University of Vienna, 1090 Vienna, Austria; 2 Department of Radiation Oncology, Medical University of Vienna, 1090 Vienna, Austria; 3 MedAustron Ion Therapy Center, 2700 Wiener Neustadt, Austria; 4 QIMP Team, Center for Medical Physics and Biomedical Engineering, Medical University of Vienna, 1090 Vienna, Austria

**Keywords:** somatostatin receptor PET, meningioma imaging, PET/CT, radiation therapy planning, Inter-observer variability

## Abstract

**Simple Summary:**

Differences in tumor segmentations between radiation oncologists is one of the largest sources of uncertainty in radiation therapy planning. This study investigated the influence of additional functional information from somatostatin receptor PET imaging on the inter observer variability in the delineation of meningioma. Further, this study assessed the usability of a simple thresholding approach for lesion delineation. It could be shown, that additional PET information was able to significantly reduce the inter observer variability. The threshold based delineation approach required a relatively low threshold value and showed only moderate agreement with the radiation oncologists.

**Abstract:**

**Aim**: The aim of this study was to assess the effects of including somatostatin receptor agonist (SSTR) PET imaging in meningioma radiotherapy planning by means of changes in inter-observer variability (IOV). Further, the possibility of using threshold-based delineation approaches for semiautomatic tumor volume definition was assessed. **Patients and Methods**: Sixteen patients with meningioma undergoing fractionated radiotherapy were delineated by five radiation oncologists. IOV was calculated by comparing each delineation to a consensus delineation, based on the simultaneous truth and performance level estimation (STAPLE) algorithm. The consensus delineation was used to adapt a threshold-based delineation, based on a maximization of the mean Dice coefficient. To test the threshold-based approach, seven patients with SSTR-positive meningioma were additionally evaluated as a validation group. **Results**: The average Dice coefficients for delineations based on MRI alone was 0.84 ± 0.12. For delineation based on MRI + PET, a significantly higher dice coefficient of 0.87 ± 0.08 was found (*p* < 0.001). The Hausdorff distance decreased from 10.96 ± 11.98 mm to 8.83 ± 12.21 mm (*p* < 0.001) when adding PET for the lesion delineation. The best threshold value for a threshold-based delineation was found to be 14.0% of the SUVmax, with an average Dice coefficient of 0.50 ± 0.19 compared to the consensus delineation. In the validation cohort, a Dice coefficient of 0.56 ± 0.29 and a Hausdorff coefficient of 27.15 ± 21.54 mm were found for the threshold-based approach. **Conclusions**: SSTR-PET added to standard imaging with CT and MRI reduces the IOV in radiotherapy planning for patients with meningioma. When using a threshold-based approach for PET-based delineation of meningioma, a relatively low threshold of 14.0% of the SUVmax was found to provide the best agreement with a consensus delineation.

## 1. Introduction

Meningiomas are, with a share of around 37%, the most common primary cerebral tumors, and are mainly treated by neurosurgery and radiotherapy (RT) [1]. magnetic resonance imaging (MRI) and computed tomography (CT) are generally established imaging modalities to delineate the tumor extension. Nevertheless, they have limitations, especially when bone structures are involved and/or the tumor is located at the skull base [2]. In such cases, molecular imaging procedures, such as positron emission tomography (PET), may provide beneficial advantages, in particular when using somatostatin receptor (SSTR) targeting radiopharmaceuticals [3,4].

With nearly 100% of meningioma cells expressing somatostatin-2-receptors (SSTR2) on their surface, meningioma can be excellently targeted by radio-labeled SSTR compounds, such as 68-gallium labeled SSTR-agonists for PET imaging [3,4]. Previous studies showed that the additional information gained by the SSTR PET did not only improve sensitivity in the diagnosis of meningiomas compared to MRI alone but also allowed a more precise tumor delineation than when using only contrast-enhanced MRI. In particular, in the case of osseous tumor infiltration or for lesions located at the skull base, as well as for therapy planning subsequent to prior therapy, SSTR PET helps to discriminate tumors from other surrounding tissues [5,6,7,8,9,10,11].

However, even though SSTR-PET seems promising for radiotherapy planning, its practical integration into clinical workflows is still a matter of debate. The target definition in radiotherapy is of utmost importance for a successful treatment. Yet, the target definition is one of the main sources of variability in the whole workflow. The additional information gained by SSTR PET is expected to facilitate tumor delineation, thus, also reducing the inter-observer variability (IOV). However, although some studies were conducted on the influence of SSTR PETs with respect to the IOV, the overall level of evidence about the asset of including SSTR PET in the therapy planning for meningioma is limited [11,12,13].

In modern radiotherapy, coherent target delineations are crucial, and a low IOV allows more reproducible treatments with fewer side effects. A reduction of IOV may be achieved by having more experienced and trained physicians, better delineation tools, and a delineation process that is guided by reliant algorithms. In particular, the use of guiding algorithms may lower the dependence of the delineation process on the physicians’ experience, and as an additional advantage, reduce the time needed for this task [14].

The aim of this study was to assess whether SSTR PET information in addition to MRI reduces the IOV of meningioma delineation compared to targeting only with MRI. In addition, this study evaluated the feasibility of a simple threshold-based delineation approach employing SSTR-PET for RT target delineation. 

## 2. Material and Methods

### 2.1. Patients

This retrospective study includes 16 patients with intracranial meningiomas, who were referred to the Department of Radiation Oncology of the Medical University of Vienna. The study cohort included 10 female and 6 male patients with an average age of 55.6 ± 15.2 (range 33–85) years. All patients had a history of recurrent meningioma after surgery. Eight patients showed more than one meningioma lesion at the time of the presented study with known histology in 23 out of 27 lesions. All lesions without histology were classified as meningioma based on their typical MRI and PET features. All patients were treated in the years 2011–2016. The number of tumor lesions of each patient varied between 1 and 6 with an average of 1.7 ± 1.3 lesions, cumulating in 27 tumors in total. Most tumors were located at the skull base, some at the convexity, and 3 lesions in the study group were located at the falx cerebri (Table 1). In addition to the 16 patients used for the IOV study and the optimization of the threshold method, a validation group of 7 independent meningioma patients was included to evaluate the threshold approach. In this group, 1.7 ± 1.3 meningioma lesions were present, cumulating in 12 lesions in total.

The study was approved by the ethical committee of the Medical University of Vienna (EK no. 1815/2019). Written informed consent was waived because of the retrospective nature of this evaluation.

### 2.2. MRI and PET/CT Imaging

Treatment planning image acquisition was performed following the institutional protocol for meningiomas using a thermoplastic mask system for patient immobilization. The protocol included a planning CT and contrast-enhanced MRI, including a T2w sequence and a T1w sequence with and without contrast enhancement. All image acquisitions were performed in the treatment position.

PET/CT imaging was done on a Siemens Biograph TPTV PET/CT system (Siemens Healthcare, Knoxville, USA). The acquisition protocol consisted of a 10 min single bed position PET acquisition 60 min after the injection of ~200 MBq of 68Ga-DOTANOC. A low-dose CT was acquired for attenuation correction and co-registration of the PET images with MRI. PET image reconstruction was done using an ordered subsets expectation-maximization algorithm (OSEM) with point spread function (PSF) correction using 4 iterations and 21 subsets into a 168 × 168 × 74 image matrix with an image dimension of 4, 4, and 5 mm, respectively. No post-reconstruction filter was applied to the images.

### 2.3. Target Delineation/Treatment Planning

All studies were transferred to iPlan RT 4.0.0 treatment planning system (BrainLab, Munich, Germany), where CT, MR, and PET/CT images for each patient were registered automatically based on bony structures and adapted manually when necessary.

Delineation was performed in two independent courses by five radiation oncologists with experience in CNS delineation. In the first course, the observers were first asked to delineate the meningioma gross tumor volume (GTV MRI) based on CT and contrast-enhanced MRI without access to the PET information. This was followed by a second delineation course where the participants had access to the PET/CT images in addition to the CT and contrast-enhanced MRI (GTV MRI + PET). The contouring was performed in a blind fashion so that no observer had any access to the structures drawn by other participants or different image data of the same patient. According to the study instructions for all observers, the same fixed window level settings had to be used, respective to the given image modality for all patients. Zooming and use of the sagittal or coronal reconstructed views were permitted and optionally used by observers.

The delineation of the validation group was done similarly, as described above by three physicians using PET + MRI information.

### 2.4. Evaluation of Inter-Observer Variability and Influence of Including PET Information

**Figure 1:**
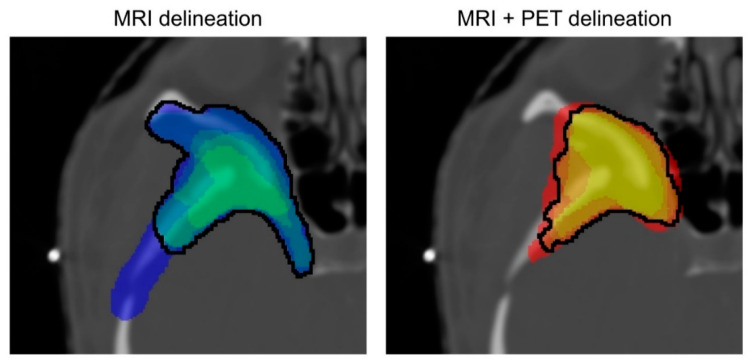
Example of the different possible consensus definitions given for the MRI and MRI + PET delineations. The color coding differentiates the amount of consensus between the physicians. The black curves outline the contours, which were created by using the STAPLE algorithm. For the MRI + PET delineation, the differences between the consensus delineations are smaller compared to the delineations based solely on the MR images.

To assess the IOV of the delineation based on MRI and MRI + PET, a consensus delineation representing the best possible delineation of the tumor for each modality was created (see Figure 1). This consensus delineation was created by employing the simultaneous truth and performance level estimation (STAPLE) algorithm. The algorithm is based on an expectation–maximization method and is commonly used in medical imaging studies. The algorithm estimates an optimal delineation by weighing each delineation of the physicians depending on an estimated performance level, as well as other factors, such as constraints on spatial homogeneity [15].

To calculate the IOV for each tumor, the MRI delineations were compared to the MRI consensus targets, by calculating the differences in volume, the Dice, as well as the Hausdorff coefficients. This step was then repeated for the delineations acquired by the addition of the PET images to the MR/CT images. 

### 2.5. Assessment of a Thresholding Approach for GTV Definition/Lesion Delineation

To test if a threshold-based delineation of the meningioma in the SSTR PET image can be used for RT planning, threshold-based tumor segmentations were compared to the STAPLE consensus delineation. Each lesion was segmented by thresholding all connected voxels in a lesion above a threshold expressed as a percentage of the maximum standardized uptake value (SUV_max_) within the lesion. This was done automatically for different thresholds from 0% to 100% in steps of 0.5% points.

For each threshold step and lesion, the Dice coefficient between the threshold-based segmentation and the consensus segmentation was calculated. The threshold, which yielded the maximum mean Dice coefficient over all lesions was selected and considered the best approximation of the GTV.

The resulting threshold was then used to delineate the validation group. These delineations were used to calculate Dice and Hausdorff coefficients between the delineations acquired with the threshold and the manual delineations based on MRI + PET.

### 2.6. Statistical Analysis

The SciPy library, version 1.6.0, as well as the Pandas library, version 1.2.2, for Python, version 3.9.5 (Python Software Foundation, Wilmington, NC, USA) were used for all statistical analyses. The volumes of the delineations of both image modalities as well as the Dice and Hausdorff coefficients were compared by a Wilcoxon signed rank test. A possible correlation between the % of SUV_max_ value and the volume of its corresponding delineation was verified with the Spearman’s rank correlation coefficient. All statistical analyses were two-sided and used 0.05 as the significance level.

## 3. Results

**Figure 2:**
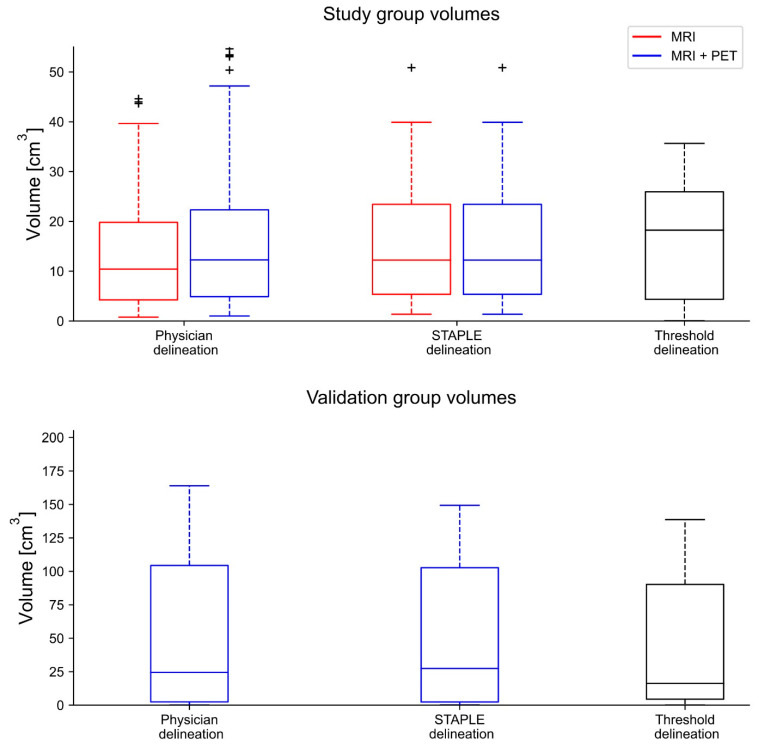
In this boxplot, the volume of each delineation method is displayed. Compared to the MRI delineations of the physicians, the MRI + PET delineations are on average bigger. The biggest volumetric outlier is found in the MRI delineation (not displayed here).

The delineated volume based solely on MRI was 13.1 ± 12.8 cm^3^ (range 0.5–95.1 cm^3^, median: 9.2 cm^3^) on average. The MRI + PET delineations were significantly larger (*p* < 0.001) with an average volume of 15.6 ± 14.8 cm^3^ (range 0.9–56.8 cm^3^, median: 11.1 cm^3^). (Figure 2). The volume of the validation cohort was 71.5 ± 113.4 cm^3^ (range 0.2–394.2 cm^3^, median: 27.4 cm^3^) based on MRI + PET images.

Average volumes of the respective physicians differed by amounts of as much as 25% between the MRI and MRI + PET delineations (Table 2). In general, physicians delineating smaller tumor volumes in the MRI-only planning also delineated smaller volumes in the MRI + PET-based planning. When looking at the percentage changes in the volume of each physician between the respective MRI and MRI + PET planning, four out of the five participating physicians had an increase in the volume of around 21% (range 17–25%), while one physician had a percentage change of −4%. This can mostly be attributed to one delineation, where this physician contoured part of the parietal bone on the MRI base planning. This area was not included by the other radiation–oncologists on MRI-only planning. The delineation based on the MRI + PET images resulted in a contour without the bone target for all delineating physicians. The physician with the highest percentage change in volume between MRI-only and PET + MRI contouring delineated the smallest volumes of all observers in MRI-only planning throughout. Using MRI + PET for delineations, the general volumes were more in agreement with those of the other physicians (Table 2).

The volumes for the consensus contours for MRI- and MRI + PET-based contouring ranged from 1.4 to 50.9 cm^3^ (average: 16.3 ± 13.8 cm^3^, median: 12.2 cm^3^) and from 1.5 to 64.0 cm^3^ (average: 18.9 ± 17.0 cm^3^, median: 14.4 cm^3^), respectively (Figure 2). The volume difference was statistically significant (*p* < 0.001).

### Inter-Observer Variability

**Figure 3:**
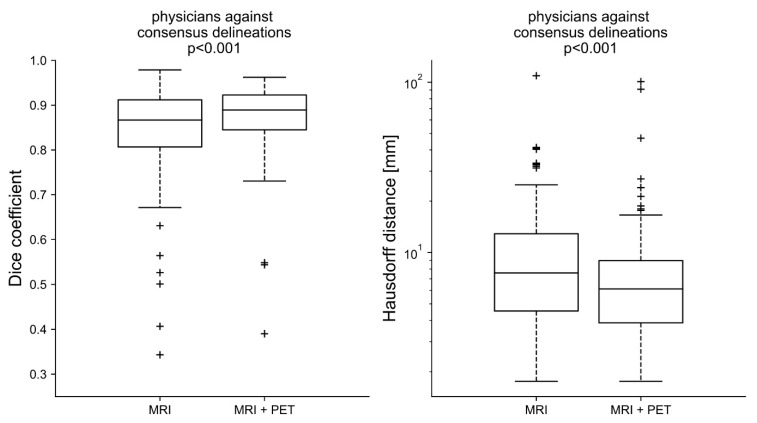
The Dice and Hausdorff coefficients were calculated for every delineation of a physician against the respective consensus delineation. The Dice coefficients for the MRI + PET modality were in closer proximity to each other and had fewer outliers (+) < 0.5, the differences in the delineations based solely on the MRI are significant (*p* < 0.001). The Hausdorff coefficients were on average also smaller for the MRI + PET approach (*p* < 0.001).

The average Dice coefficient for the MRI delineations against the MRI consensus contour was 0.84 ± 0.12 (range: 0.22–0.98, median: 0.87), whereas the average Dice coefficient for the MRI + PET delineations against the respective consensus contour was 0.87 ± 0.08 (range: 0.39–0.96, median: 0.89). The respective Hausdorff distances were 10.96 ± 11.98 mm (range: 1.76–109.25 mm, median: 7.59 mm) for MRI-only and 8.83 ± 12.21 mm (range: 1.76–100.83 mm, median: 6.11 mm) for the MRI + PET delineations (Figure 3). MRI-only and MRI + PET delineations were statistically significantly different for Dice coefficients (*p* < 0.001) as well as for the Hausdorff distance (*p* = 0.001).

#### Threshold-Based Delineation

**Figure 4:**
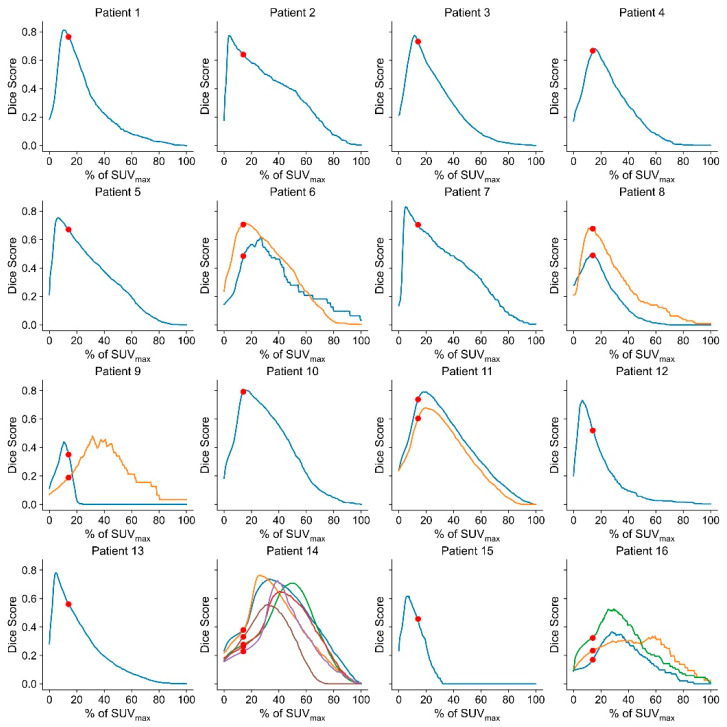
Dice score plotted against the threshold value % of SUVmax for every patient. The red dots mark the % of SUVmax, which maximized the overall Dice coefficient. For Patients 6, 8, 11, 14, and 16, multiple lesions were present (depicted as different lines), which were treated independently for the calculation of the threshold.

As can be seen in Figure 4, the behavior of the Dice coefficient in relation to the used SUV threshold was similar for most patients and tumors. By varying the threshold value, in a way that the overall Dice coefficient was maximized, a threshold value of 14.0 of the SUV_max_ was found. This value was then applied for delineation and resulted in a Dice coefficient of 0.50 ± 0.19 (range: 0.05–0.79) against the consensus delineation. The average volume of the threshold delineations was 20.9 ± 22.3 cm^3^ (range: 0.1–98.1 cm^3^, median: 18.2 cm^3^). No statistically significant correlation between the threshold (% of SUV_max_) and the volume was found (*p* = 0.11).

By comparing the threshold-based to the physician-based delineations of the validation group, a Dice coefficient of 0.56 ± 0.29 (range: 0.03–0.88, median: 0.60) and a Hausdorff coefficient of 27.15 ± 21.54 mm (range: 3.60–63.97 mm, median: 19.97 mm) was obtained. The average volume of the threshold delineations of the control group was 62.9 ± 96.9 cm^3^ (range: 0.1–329.4 cm^3^, median: 16.3 cm^3^). These volumes were not significantly smaller than the STAPLE-based ones (*p* = 0.52).

## 4. Discussion

Similar to previous studies, it was observed that additional information provided by SSTR PET changed the treatment planning tumor volumes significantly [8,9,11,13,16]. Although the average metabolic tumor volume was increased with this additional image information, there were fewer outliers, such as an MRI contour with a substantially overestimated volume of up to 95.1 cm^3^ in one case of this study. Here, the tumor volume was in much closer proximity to the remaining tumor delineation volumes drawn by the other physicians after adding PET information to the treatment planning. The changes in tumor volumes after the addition of PET information were not uniform across physicians. While for most physicians, the added image modality led to an increase in volume, a general decrease in delineation volume for one physician was observed. This may have been due to the different experience levels of the radiation oncologists in meningioma delineation with consecutive under- or overestimated tumor volume compared to the consensus delineation based on MRI images alone.

In general, IOV was reduced significantly when adding PET information, which is in agreement with former studies by MacLean et al. [12] and Perlow et al. [11]. The reduction in IOV was exemplified by the higher Dice coefficient found for the MRI + PET-based planning compared to the Dice coefficient found for MRI-only-based planning. The same was also true for the Hausdorff coefficient, even though the highest Hausdorff coefficient was found for an MRI + PET-based delineation. This outlier was due to a faulty delineation, where some voxels outside the actual target volume were erroneously included in the GTV of the treatment planning.

Using the consensus delineations, it was possible to define an appropriate threshold of 14.0% of SUV_max_ for the threshold-based target delineation. In contrast to threshold-based approaches for other tumors, such as lung tumors for which a threshold value of 42% was found to represent tumor extent best [17], the ideal threshold value was rather low. This observation was attributed to the high specificity of SSTR PET with a high target uptake and almost negligible physiological uptake in the brain [17].

In general, a dice coefficient of 0.50 +/− 0.19 against a consensus delineation (as found for the threshold-based meningioma segmentation) can be regarded as an indicator of a moderate performance of the simple threshold-based approach. This Dice coefficient is rather low compared to values found in other publications, presenting (semi)automatic delineation approaches for gliomas and lung carcinomas. For these tumor types, Dice coefficients between 0.58 and 0.82 were described using simple threshold methods, as well as more sophisticated approaches [18,19,20,21,22]. This was further confirmed in the validation group yielding an average Dice coefficient of 0.56 between the threshold approach and a consensus delineation.

Thresholding on PET might be challenged by the limited resolution of PET compared to CT and MRI. Approximations between voxels need to be addressed, as a simple threshold approach might under- or overestimate the real tumor volume in cases of relatively small lesions. This can also explain the delineations of patients 14 and 16, where the tumors presented relatively slim and were attached to the skull in diagonal orientations in relation to the pixel grids. This may lead to volatile Dice coefficients and the need for adapted threshold values. A further challenge involves the insufficient discrimination of tumors from the pituitary gland, which exhibits strong physiological tracer uptake at SSTR PET [16]. In one patient, this led to the erroneous inclusion of bilateral areas, and parts of the pituitary gland itself at threshold-based contouring, an error not incurred by delineating physicians. Therefore, a threshold-based approach as presented in this study can serve as an initial contouring proposal of a meningioma lesion but needs to be checked and adjusted by a radiation oncologist to avoid such erroneous delineations.

As for the limitations of this study, it needs to be noted that it was based on data from a single institution. Therefore, the influence of a larger patient cohort and institutional differences in imaging and delineation protocols remains to be determined. Moreover, the PET reconstruction protocol might affect the ideal value of the threshold for delineation, which was outside the scope of the present study. The lesion appearance and distance (to each other) for each lesion counted as individuals requiring their own delineations. Therefore, it is believed that the lesions can be treated as individual lesions within this study. Although there is a need in the future to investigate the different magnitudes of the effects of SSTR PET on IOV, with more focus, and with a higher number of included lesions in patients with primary or recurrent meningioma with bone and dural infiltration, our data show that SSTR-based information has a positive effect on IOV in radiotherapy planning. Finally, the best delineation of a lesion includes the actual extent of the tumor. This might not be objectively assessable by only current radiological methods used. Therefore, a tumor delineation based on the consensus of multiple radiation oncologists was performed in this study to produce the best estimate of an ideal delineation. 

## 5. Conclusions

SSTR PET imaging provides important additional information and reduces the IOV for meningioma radiotherapy planning. The metabolic tumor information leads to the inclusion of additional meningioma tissue not recognized on MRI images alone and to the exclusion of areas where tumor masses are interpreted solely based on MRI information. Further, the findings within this work indicate that the performance of a simple thresholding approach for lesion delineation based on SSTR PET is moderate.

## Figures and Tables

**Table 1:** Summary of the patient collective used in this study. * Unknown: histologies of these lesions were unknown regarding subtype and/or grading and defined as meningioma based on typical MRI and PET features.

	Study Group	Validation Group
Number of patients	16	7
Age (*p* = 0.24)	55.6 ± 15.2	61.4 ± 12.5
Sex: w/m (*p* = 0.30)	10/6	6/1
Number of lesions	27	7
Number of lesions per patient (*p* = 0.97)	1.7 ± 1.3 (range: 1–6)	1.7 ± 1.3 (range: 1–4)
Location of the lesions	Skull base: 19× Falx: 3× Calvaria: 5×	Skull base: 10× Calvaria: 2×
Meningioma subtype	Meningothelial: 11 lesions Clear cell: 1 lesion Secretory: 1 lesion Transitional: 1 lesion Atypical: 2 lesions Anaplastic: 5 lesions Unknown: 6 lesions	Unknown: 12 lesions
Grading (WHO 2016)	I: 13 lesions II: 5 lesions III: 5 lesions Unknown *: 4 lesions	I: 5 lesions Unknown *: 7 lesions

**Table 2:** Summary of the volumes of the delineations by the respective physicians. The percentage change in volume between the delineations based solely on MRI and MRI + PET images is given in the last column.

Physician	Modality	Average Volume (cm^3^)	Standard Deviation (cm^3^)	Median Volume (cm^3^)	Minimum Volume (cm^3^)	Maximum Volume (cm^3^)	% Change in Volume between Modalities
CLC	MRI	11.3	8.9	9.0	0.8	29.4	25.0
	MRI + PET	15.1	14.9	10.2	1.1	56.8	
CW	MRI	13.2	10.8	10.5	0.6	39.1	23.5
	MRI + PET	17.3	16.0	11.4	1.1	56.0	
HH	MRI	16.1	19.5	9.2	1.0	95.1	−3.9
	MRI + PET	15.5	14.9	13.2	1.3	53.1	
SK	MRI	13.2	11.6	10.2	0.8	43.8	16.5
	MRI + PET	15.8	15.1	12.8	1.0	53.4	
ULB	MRI	11.7	10.8	8.9	0.5	44.0	18.0
	MRI + PET	14.2	14.1	10.0	0.9	54.7	

## Data Availability

The image data used in this study are medical data, which must not be shared with externals according local regulations. Any reuse of the data requires an additional ethics approval by the local ethics committee. For reasonable requests, please contact the corresponding author.
